# Supplementary material for: Transmission of SARS-CoV-2 before and after symptom onset: impact of nonpharmaceutical interventions in China
Source: Eur J Epidemiol. 2021 Apr 21;36(4):429–39. doi: 10.1007/s10654-021-00746-4 (PMC8058147; doi:10.1007/s10654-021-00746-4)
Supplement: Supplementary file 1 — Supplementary file1 (PDF 1388 kb) [file 10654_2021_746_MOESM1_ESM.pdf]

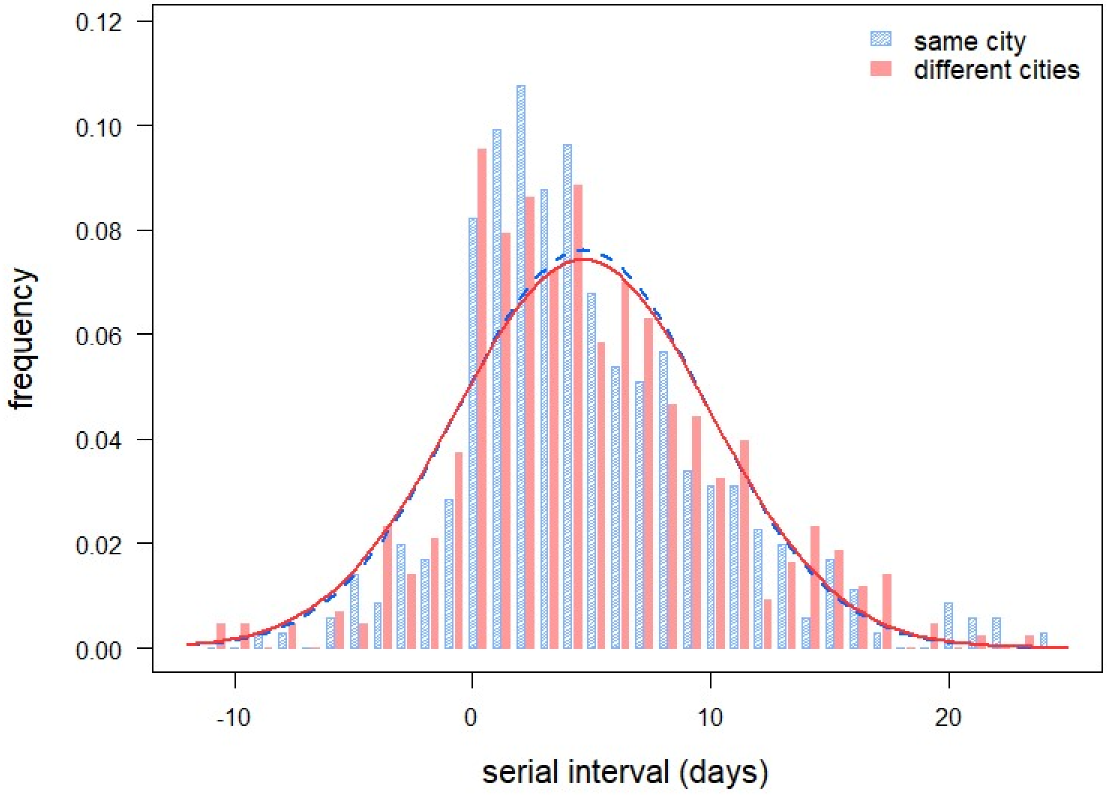

Fig. S1 Serial intervals and fitted normal distributions for case pairs infected in the same city (blue hatched bars, dashed blue line) and different cities (red solid bars, red solid line).

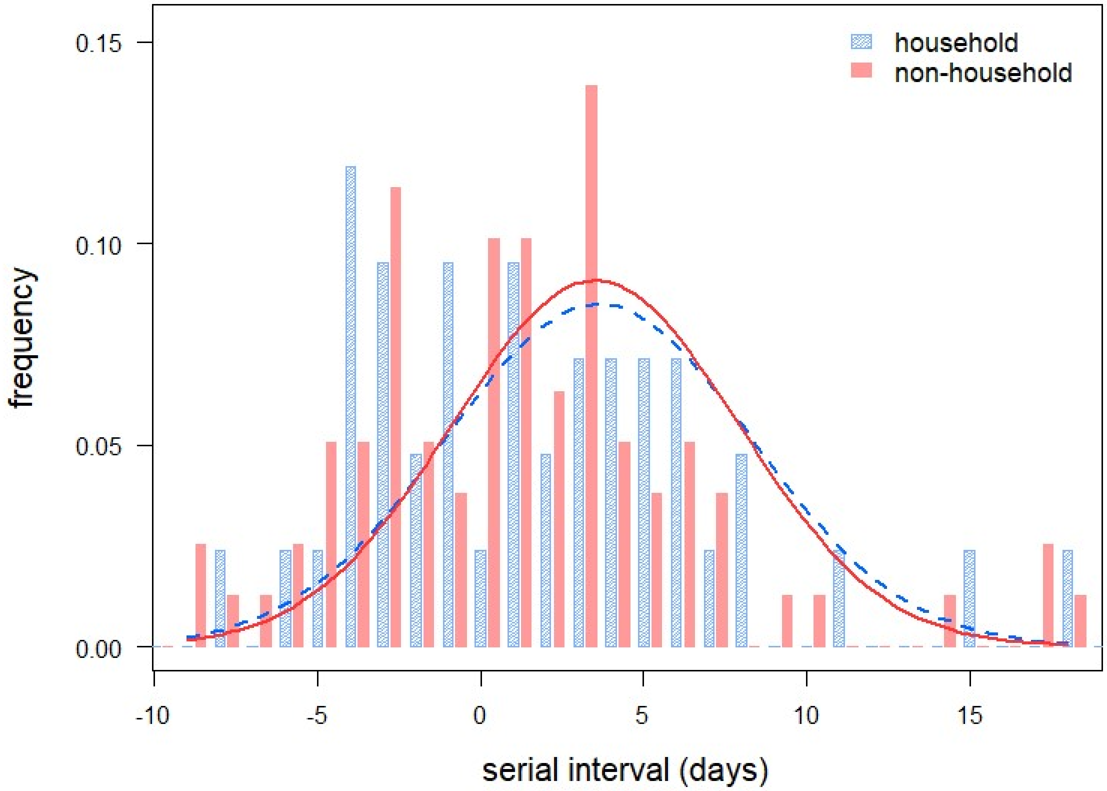

Fig. S2 Serial intervals and fitted normal distributions for case pairs with household contact (blue hatched bars, dashed blue line) or non-household contact (red solid bars, red solid line).

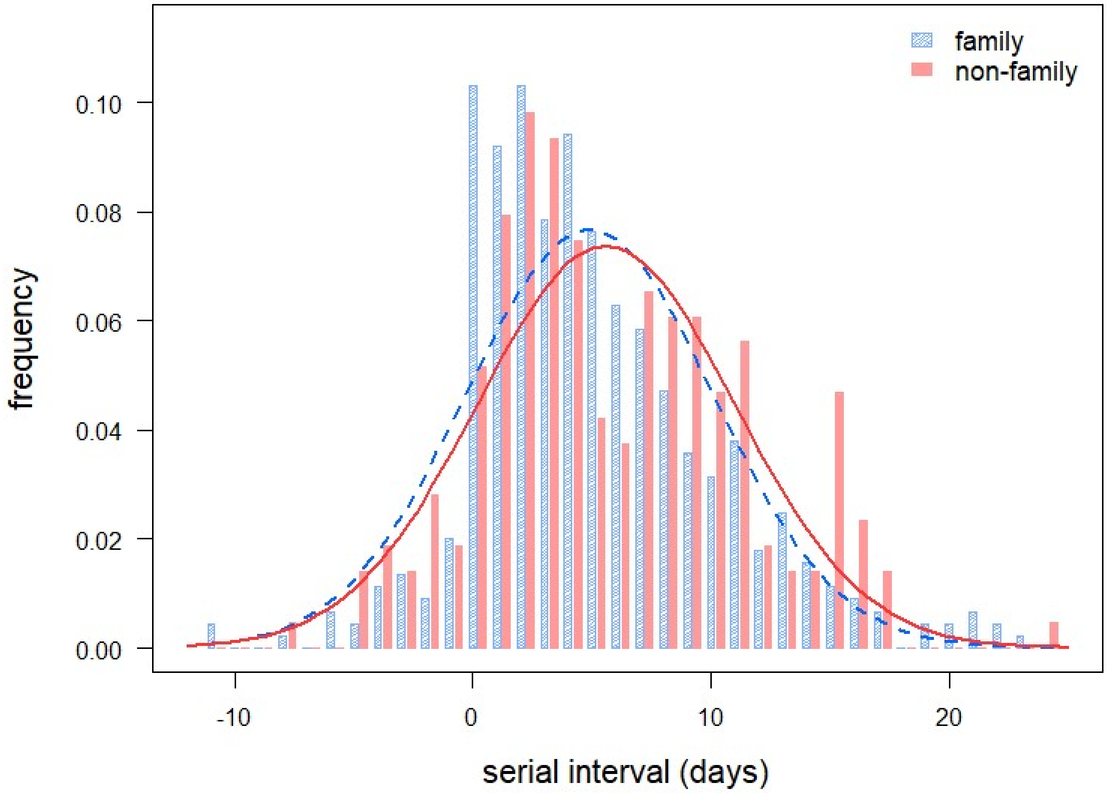

Fig. S3 Serial intervals and fitted normal distributions for case pairs with family relationships (blue hatched bars, dashed blue line) or non-family relationships (red solid bars, red solid line).

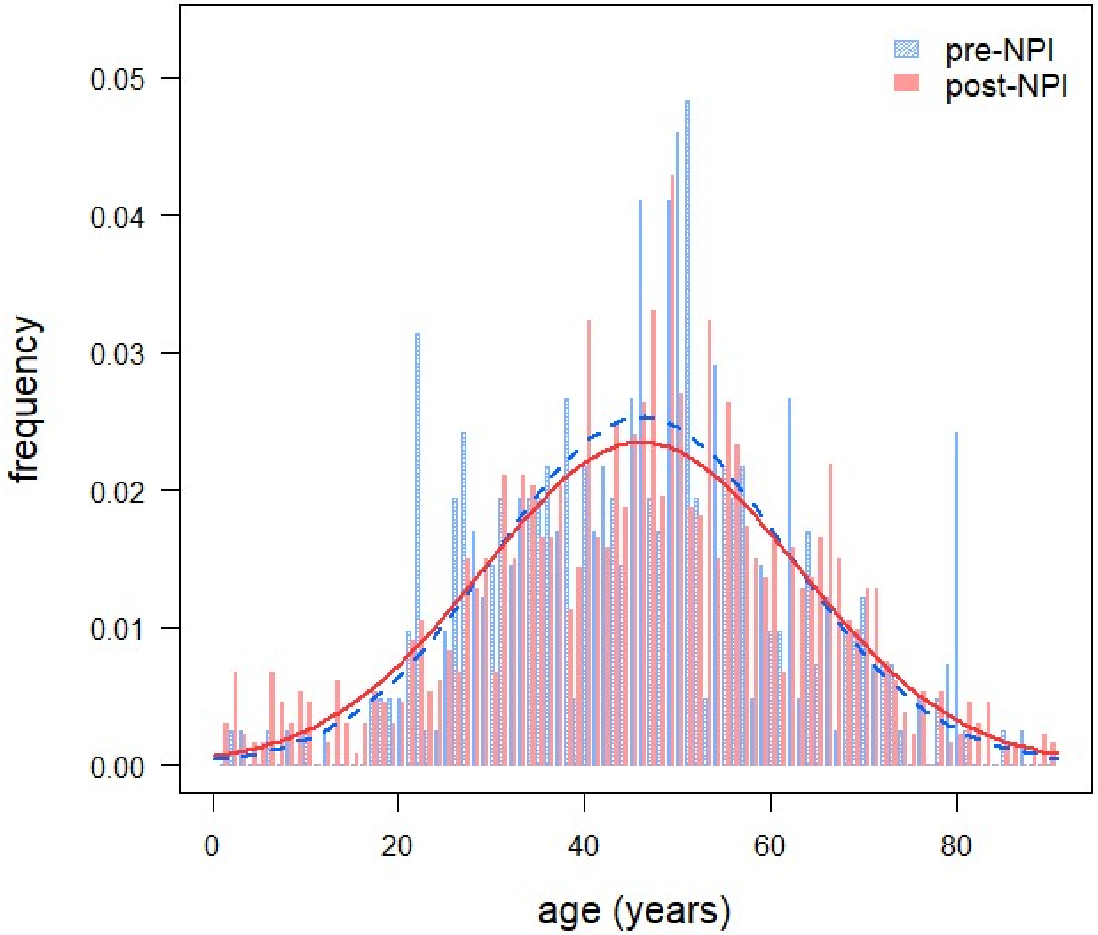

Fig. S4 Ages in the pre- and post-NPI time periods, with fitted normal distributions. Pre-NPI, hatched blue bars and dashed blue line; post-NPI, solid red bars and solid red line.

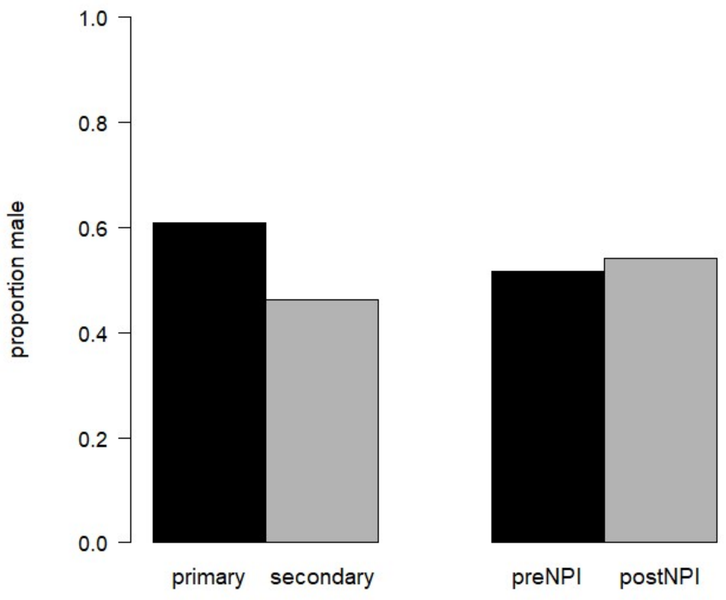

Fig. S5 Fraction of cases reported as male, stratified by case position (primary or secondary) and time period (pre-NPI or post-NPI).

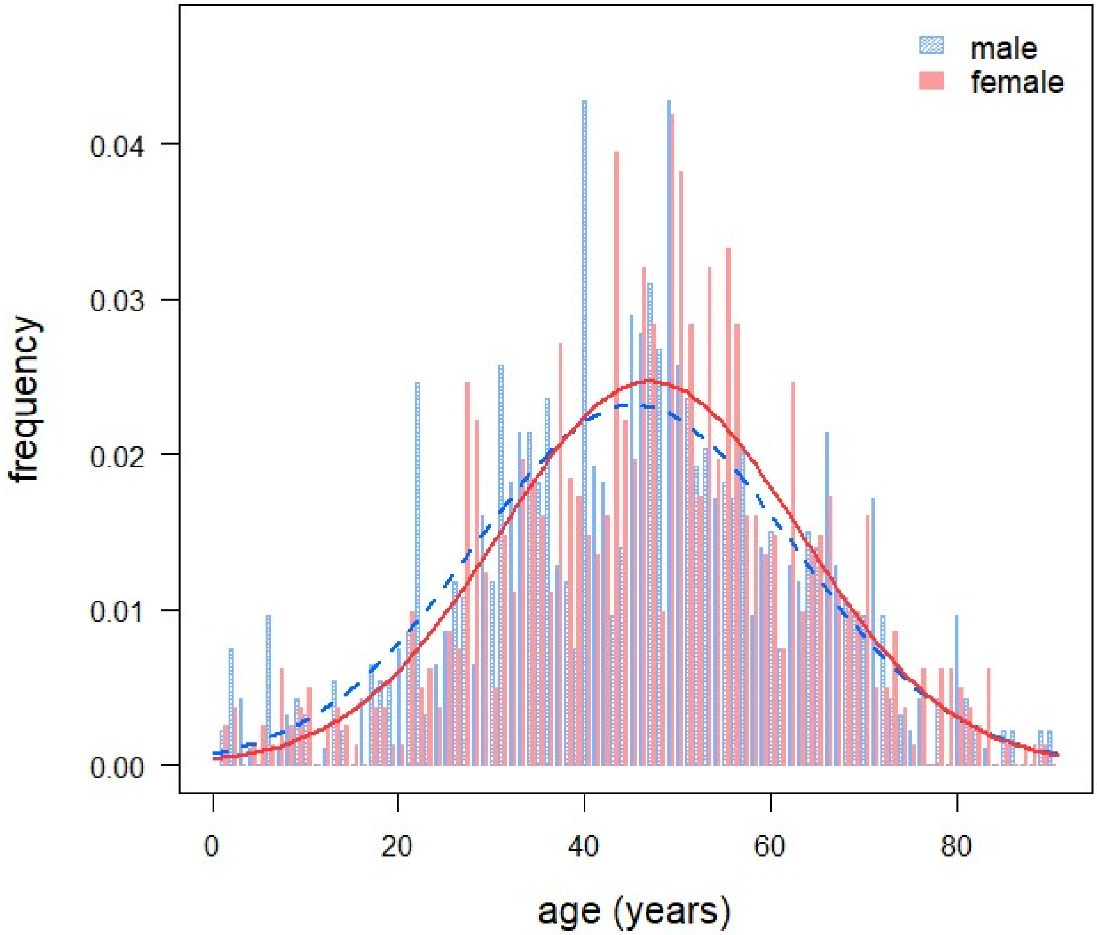

Fig. S6 Ages among male and female cases, with fitted normal distributions. Male, hatched blue bars and dashed blue line; female, solid red bars and solid red line.

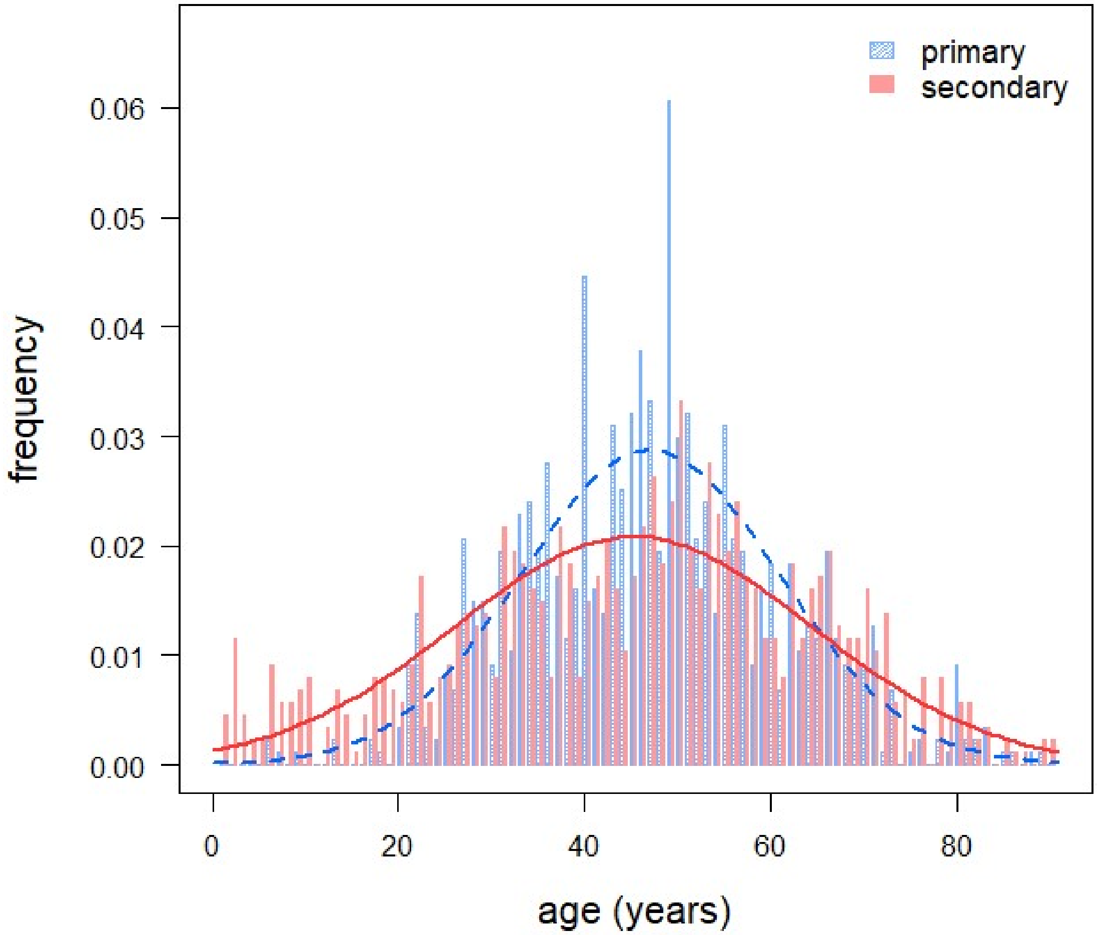

Fig. S7 Ages among primary and secondary cases, with fitted normal distributions. Primary cases, hatched blue bars and dashed blue line; secondary cases, solid red bars and solid red line.

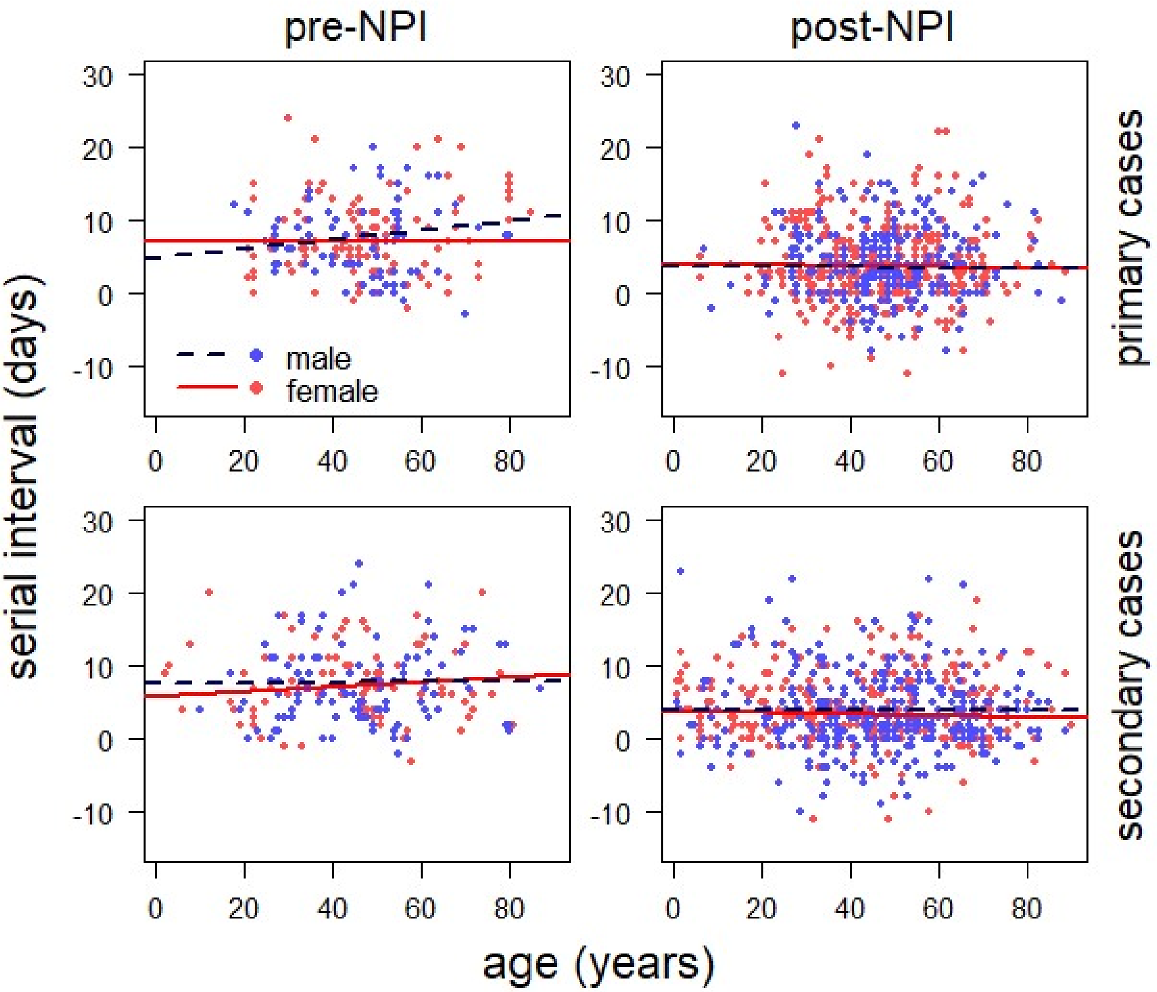

Fig. S8 Serial intervals as a function of age and sex, with regression lines (serial interval vs age) for each sex. Male, blue points and dashed blue lines; female, red points and solid red lines. Data are stratified by time period (pre-NPI, left; post-NPI, right) and case position (primary cases, top; secondary cases, bottom).

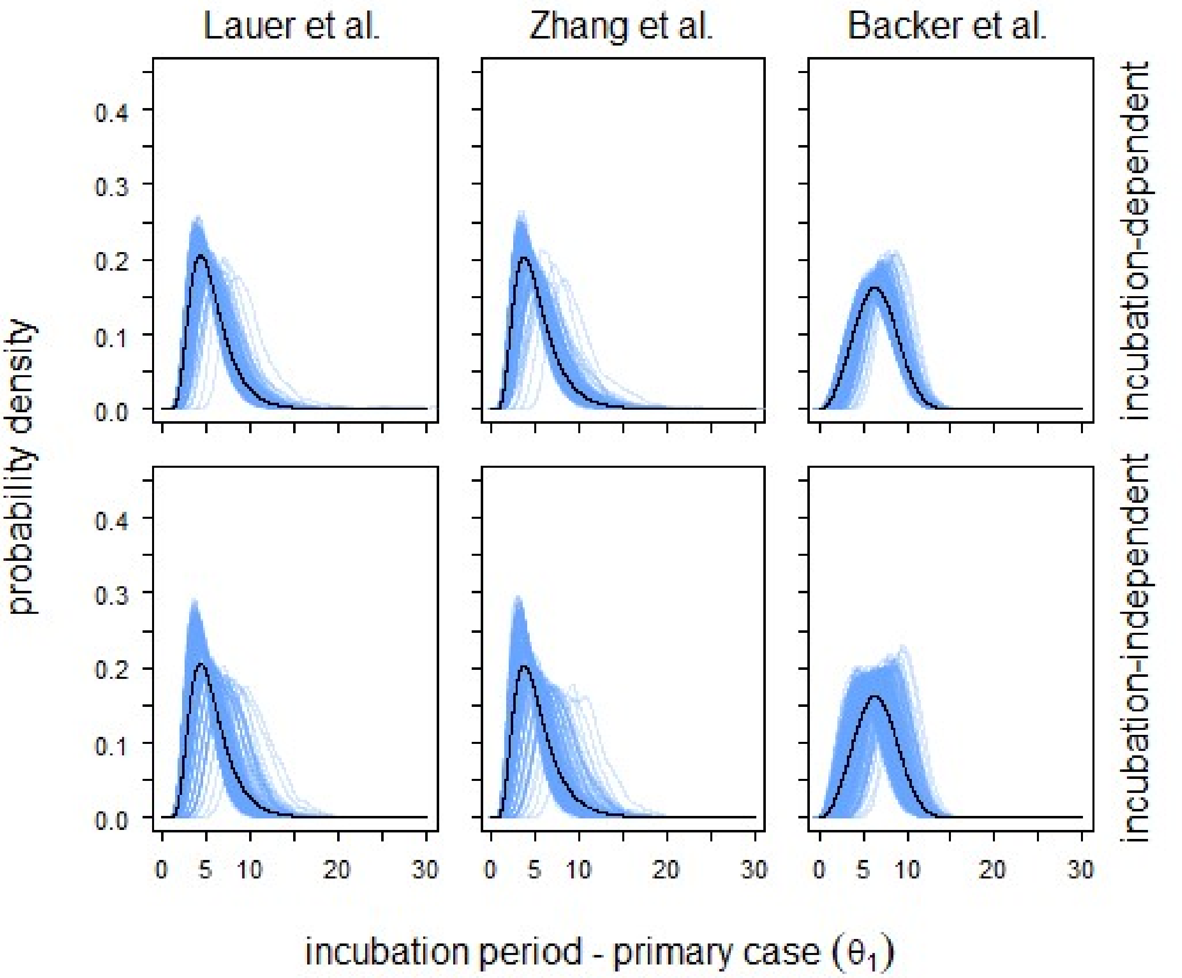

Fig. S9 Overlaid posterior distributions for the incubation period of the primary case (θ_1) of each case pair in the pre-NPI period. Black lines show the incubation period prior for each analysis.

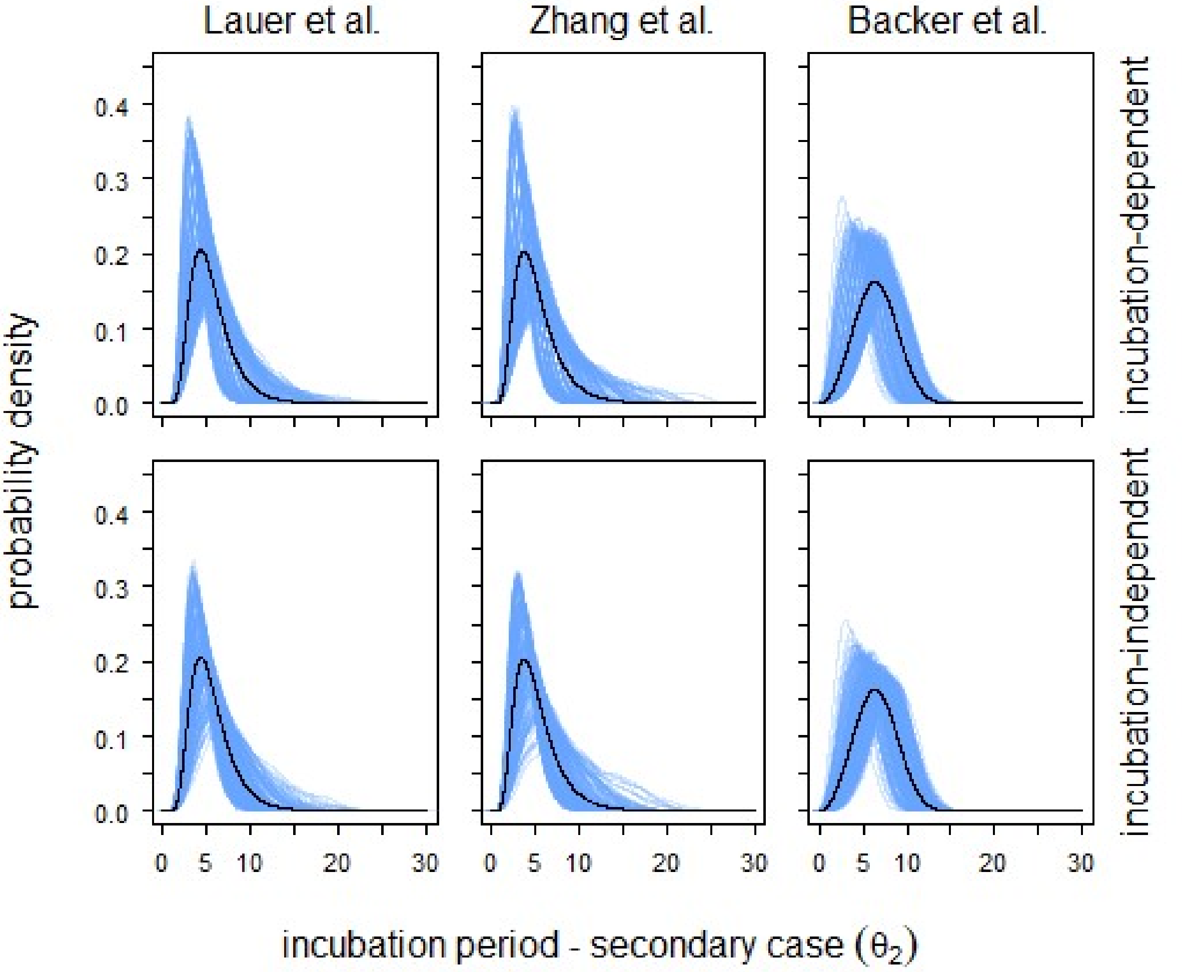

Fig. S10 Overlaid posterior distributions for the incubation period of the secondary case (θ_2) of each case pair in the pre-NPI period. Black lines show the incubation period prior for each analysis.

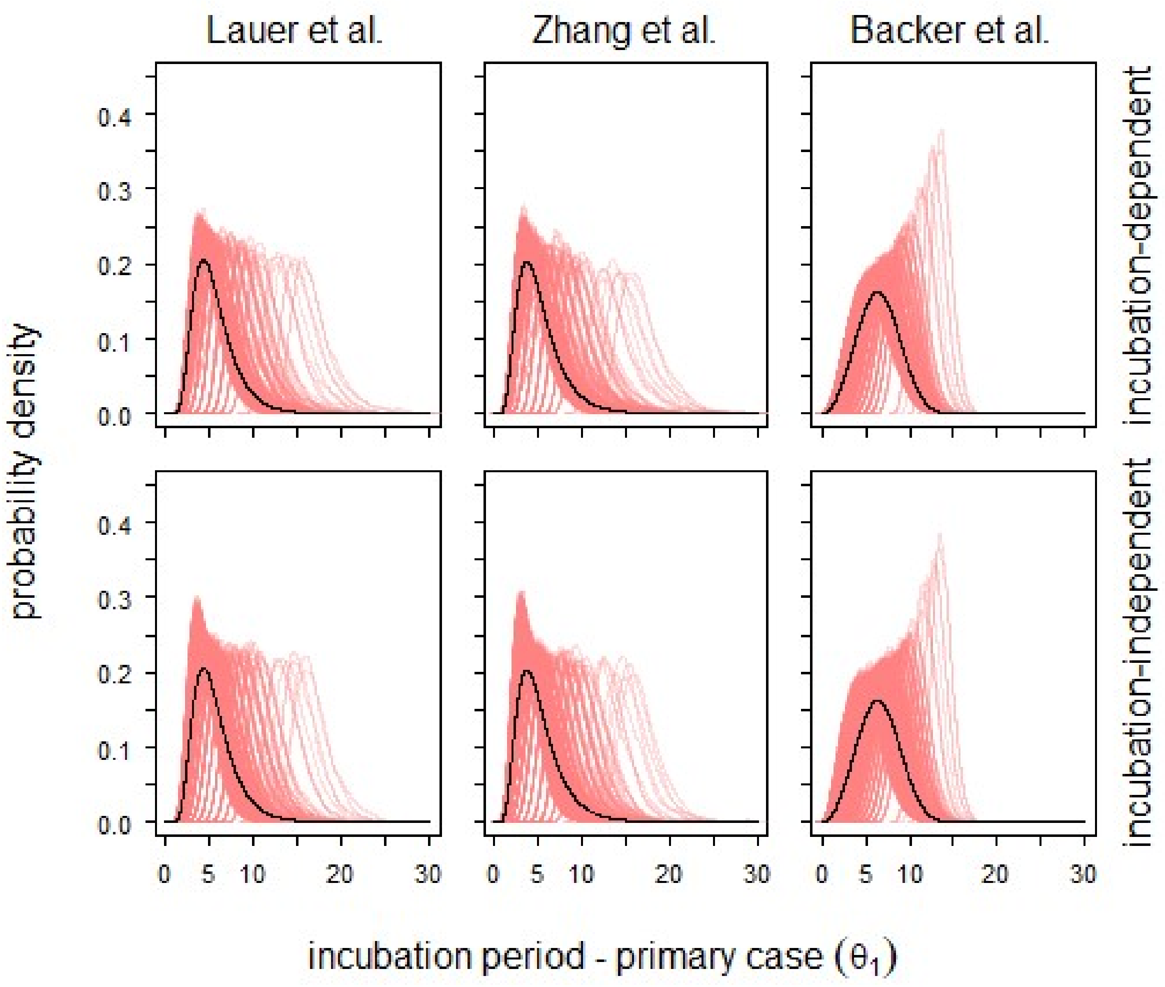

Fig. S11 Overlaid posterior distributions for the incubation period of the primary case (θ_1) of each case pair in the post-NPI period. Black lines show the incubation period prior for each analysis.

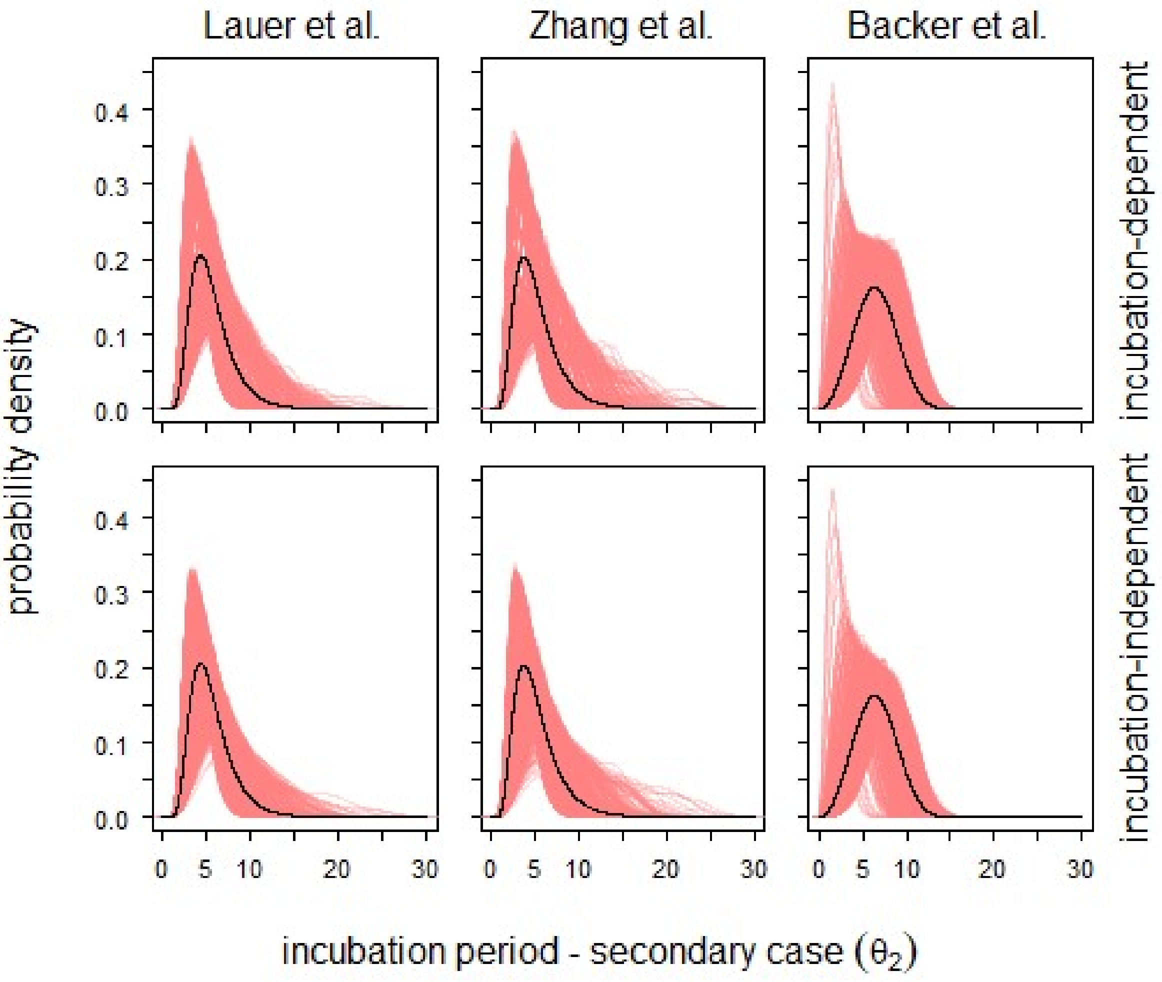

Fig. S12 Overlaid posterior distributions for the incubation period of the secondary case (θ_2) of each case pair in the post-NPI period. Black lines show the incubation period prior for each analysis.

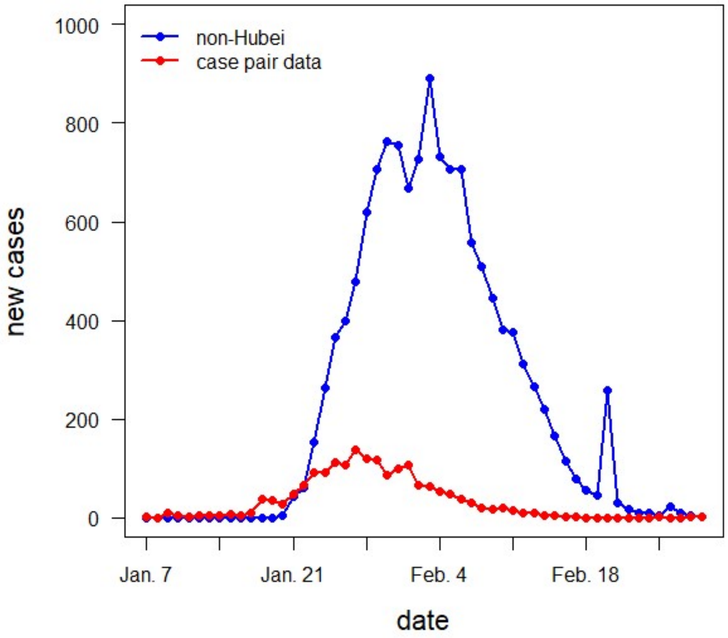

Fig. S13 Case incidence based on case pairs (red) and non-Hubei cases (blue).

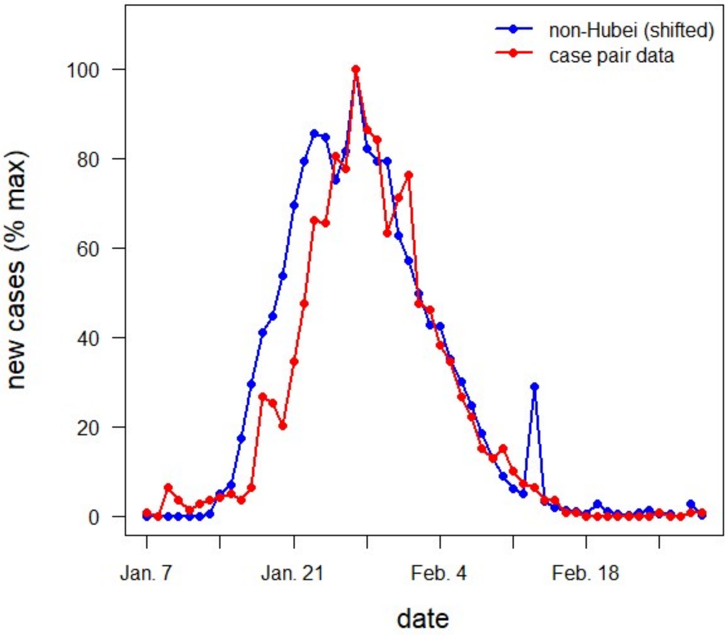

Fig. S14 Case incidence based on case pairs (red) and non-Hubei cases (blue), with both curves scaled to their respective maximums and the non-Hubei incidence curve shifted 7 days to the left.

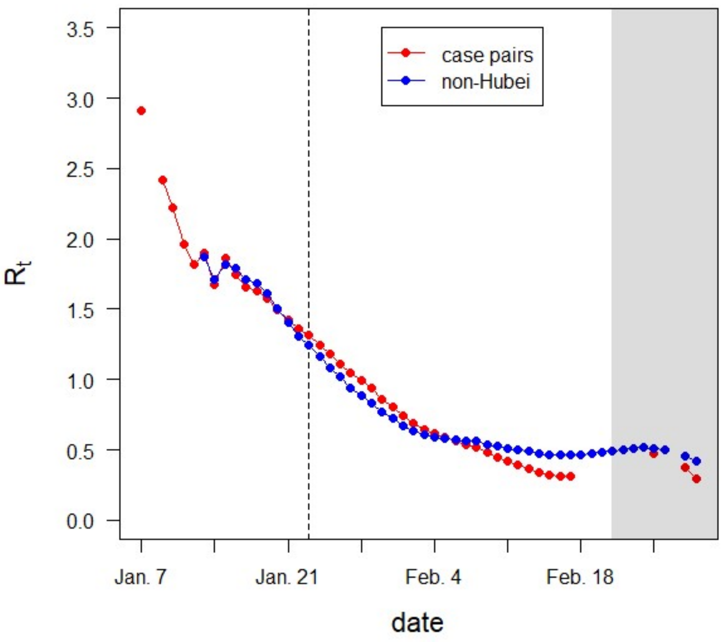

Fig. S15 Daily estimates of R_t based on case pair incidence data (red) and non-Hubei incidence data (blue). Dashed vertical line marks January 23, the beginning of the Wuhan lockdown and the start of NPI rollout across the country. Gray shading covers region in which R_t is likely to be underestimated due to right-truncation.

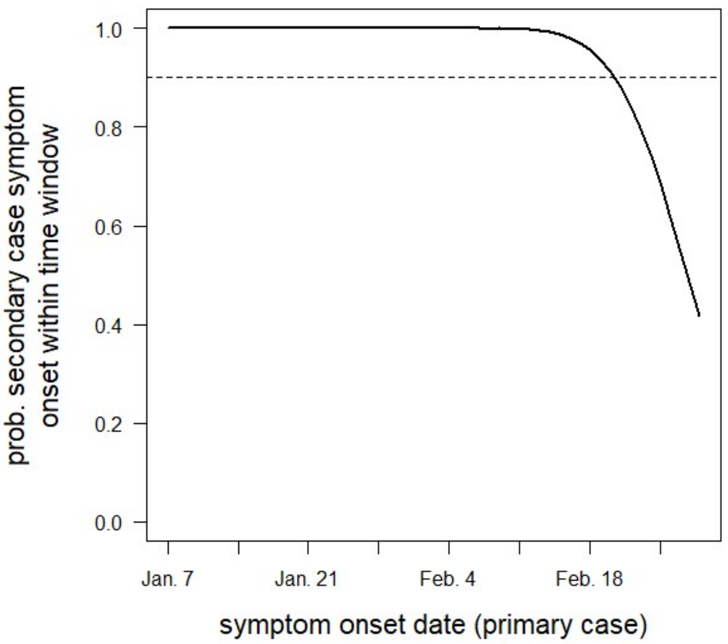

Fig. S16 Probability of a secondary case developing symptoms by $t_{\max}$ (Feb. 29) vs. time of primary case symptom onset. Dashed line shows probability cutoff (0.9).

	Primary infection location				
**Secondary infection location**		**Hubei**	**Non-Hubei**	**Unknown**	**Marginal**
	**Hubei**	62 (7.1%)	3 (0.3%)	1 (0.1%)	66 (7.6%)
	**Non-Hubei**	324 (37.1%)	356 (40.8%)	55 (6.3%)	735 (84.2%)
	**Unknown**	11 (1.3%)	8 (0.9%)	53 (6.1%)	72 (8.2%)
	**Marginal**	397 (45.5%)	367 (42.0%)	109 (12.5%)	873 (100%)

Table S1 Chinese provinces in which primary and secondary cases were presumed to be infected.

Model parameter	Prior family	Prior parameters	Source
α	Uniform	$a = 0$<br>$b = 100$	n/a
β	Uniform	$a = 0$<br>$b = 100$	n/a
θ	Lognormal	$\mu = 1.621$<br>$\sigma = 0.418$	Lauer et al. [1]
θ	Lognormal	$\mu = 1.540$<br>$\sigma = 0.470$	Zhang et al. [2]
θ	Weibull	$\lambda = 7.263$<br>$k = 3.009$	Backer et al. [3]

Table S2 Prior distributions for MCMC parameter estimation.

Time period	Generation interval model	Incubation period prior	α		β		DIC
			mean	*95% CI*	*mean*	*95% CI*	
Pre-NPI	incubation-independent	Lauer et al.	3.71	(2.57, 4.99)	0.495	(0.344, 0.666)	2899
		Zhang et al.	4.05	(2.66, 6.68)	0.541	(0.355, 0.886)	2916
		Backer et al.	3.99	(2.72, 5.71)	0.527	(0.362, 0.745)	2983
	incubation-dependent	Lauer et al.	2.87	(2.12, 3.83)	2.09	(1.49, 2.86)	2932
		Zhang et al.	2.98	(2.23, 3.87)	2.04	(1.48, 2.73)	2955
		Backer et al.	3.03	(2.22, 4.00)	2.58	(1.85, 3.41)	3005
Post-NPI	incubation-independent	Lauer et al.	1.55	(1.27, 1.91)	0.398	(0.325, 0.490)	8896
		Zhang et al.	1.65	(1.31, 2.06)	0.430	(0.338, 0.543)	8923
		Backer et al.	1.51	(1.23, 1.82)	0.382	(0.313, 0.459)	9190
	incubation-dependent	Lauer et al.	1.25	(1.05, 1.49)	1.73	(1.43, 2.09)	8971
		Zhang et al.	1.34	(1.12, 1.60)	1.77	(1.43, 2.15)	9014
		Backer et al.	1.24	(1.05, 1.45)	1.99	(1.66, 2.37)	9222

Table S3 Posterior means and 95% credible intervals for the parameters of the generation interval distribution, plus deviance information criterion (DIC) estimates for each model and each time period.

Time period	Generation interval model	Incubation period prior	mean		sd	
			mean	*95% CI*	*mean*	*95% CI*
Pre-NPI	incubation-independent	Lauer et al.	7.50	(6.81, 8.20)	3.95	(3.32, 4.74)
		Zhang et al.	7.49	(6.80, 8.22)	3.83	(2.90, 4.65)
		Backer et al.	7.58	(6.90, 8.28)	3.86	(3.18, 4.61)
	incubation-dependent	Lauer et al.	7.63	(6.89, 8.42)	5.99	(5.14, 6.97)
		Zhang et al.	7.62	(6.87, 8.45)	6.28	(5.41, 7.24)
		Backer et al.	7.63	(6.94, 8.38)	5.48	(4.76, 6.34)
Post-NPI	incubation-independent	Lauer et al.	3.90	(3.59, 4.24)	3.15	(2.78, 3.53)
		Zhang et al.	3.84	(3.49, 4.18)	3.01	(2.60, 3.43)
		Backer et al.	3.95	(3.63, 4.30)	3.23	(2.89, 3.61)
	incubation-dependent	Lauer et al.	3.99	(3.66, 4.34)	4.28	(3.83, 4.77)
		Zhang et al.	3.96	(3.62, 4.32)	4.31	(3.84, 4.85)
		Backer et al.	4.04	(3.71, 4.38)	4.14	(3.73, 4.59)

Table S4 Posterior means and 95% credible intervals for the mean and standard deviation of the generation interval distribution.

Generation interval model	Incubation period prior	Pre-NPI		Post-NPI	
		mean	*95% CI*	*mean*	*95% CI*
incubation-independent	Lauer et al.	34.4%	(28.3%, 41.3%)	71.0%	(67.6%, 74.2%)
	Zhang et al.	30.7%	(24.0%, 37.7%)	68.1%	(64.5%, 71.7%)
	Backer et al.	43.7%	(37.1%, 50.4%)	77.5%	(74.5%, 80.3%)
incubation-dependent	Lauer et al.	37.8%	(31.4%, 44.5%)	75.1%	(71.8%, 78.3%)
	Zhang et al.	34.1%	(27.7%, 40.8%)	73.2%	(69.5%, 76.8%)
	Backer et al.	47.0%	(40.2%, 53.7%)	80.6%	(77.6%, 83.5%)

Table S5 Posterior means and 95% credible interavls for the relative frequency of presymptomatic transmission during the pre-NPI and post-NPI periods.

Generation interval model	Incubation period prior	presymptomatic transmission		transmission post-symptom onset	
		mean	*95% CI*	*mean*	*95% CI*
incubation-independent	Lauer et al.	-15.5%	(-30.6%, +2.56%)	-82.0%	(-84.5%, -79.0%)
	Zhang et al.	-8.62%	(-27.0%, +16.3%)	-81.3%	(-84.0%, -78.3%)
	Backer et al.	-27.5%	(-37.9%, -15.1%)	-83.7%	(-86.3%, -80.7%)
incubation-dependent	Lauer et al.	-18.7%	(-31.8%, -2.11%)	-83.7%	(-86.3%, -80.7%)
	Zhang et al.	-12.0%	(-27.6%, +7.51%)	-83.5%	(-86.1%, -80.5%)
	Backer et al.	-29.9%	(-39.4%, -18.2%)	-85.1%	(-87.9%, -81.9%)

Table S6 Posterior means and 95% credible intervals for the % change in the absolute frequency of presymptomatic transmission and transmission post-symptom onset after the introduction of NPIs.

Transmission type	Pre-NPI	Post-NPI
Household	7	35
Non-household	26	53
Family	81	365
Non-family	71	143
Infected in different cities	126	303
Infected in same city	61	292

Table S7 Numbers of six types of transmission events: attributable to household contact; attributable to non-household contact; between family members; between non-family members; primary and secondary cases infected in different cities; primary and secondary cases infected in the same city. Contact type, relationship, and infection location data were not available for all case pairs, so the totals for household/non-household (121), family/non-family (660), and imported/non-imported (782) are different from one another and less than the total number of case pairs (873).

Applying deviance information criteria (DIC) to models of presymptomatic transmission of SARS-CoV-2

A comprehensive guide to deviance information criteria (DIC) for missing data models exists in the paper by Celeux et al; here, we introduce only those definitions and concepts applicable to our models. The first part is devoted to DIC in general, while the second part shows how DIC is calculated for our models in particular.

Part I: Theory

Introduction

Let $f(\mathbf{y}|\Theta)$ be the likelihood of observing some data $\mathbf{y}$, given a model f with parameter(s) Θ.

The *deviance* of model f with parameters Θ is defined as

$$D(\Theta) = -2\log f(\mathbf{y}|\Theta) + 2\log h(\mathbf{y})$$

where $h(\mathbf{y})$ is a function of the data alone, i.e. it does not depend on Θ.

The deviance information criterion, or DIC, is defined by the following expression:

$$\text{DIC} = \overline{D(\Theta)} + p_D$$

$\overline{D(\Theta)}$ is the posterior mean deviance, a Bayesian measure of model fit, which is found by taking the expectation of $D(\Theta)$ over the posterior distribution of Θ:

$$\overline{D(\Theta)} = E_\Theta[-2\log f(\mathbf{y}|\Theta)|\mathbf{y}] + 2\log h(\mathbf{y})$$

p_D is the effective number of parameters, a measure of model complexity, and is given by

$$p_D = \overline{D(\Theta)} - D(\tilde{\Theta})$$

where $\tilde{\Theta}$ is an estimate for Θ given $\mathbf{y}$, such as the posterior mean $E_\Theta[\Theta|\mathbf{y}]$.

We can therefore rewrite DIC as follows:

$$\text{DIC} = 2\overline{D(\Theta)} - D(\tilde{\Theta})$$

$$= -4E_\Theta[\log f(\mathbf{y}|\Theta)|\mathbf{y}] + 2\log f(\mathbf{y}|\tilde{\Theta}) + 2\log h(\mathbf{y})$$

Since $h(\mathbf{y})$ does not depend on the model or parameters, it is irrelevant for model comparison; we can therefore set $h(\mathbf{y}) = 1$ which makes $2\log h(\mathbf{y}) = 0$, simplifying the expression for DIC to

$$\text{DIC} = -4E_\Theta[\log f(\mathbf{y}|\Theta)|\mathbf{y}] + 2\log f(\mathbf{y}|\tilde{\Theta})$$

Estimating DIC from Markov chain Monte Carlo (MCMC)

DIC can be readily applied to model fitting by MCMC because the posterior distribution of Θ is approximated by the post-convergence Markov chain. As long as the likelihood function $f(\mathbf{y}|\Theta)$ is available in closed form, the posterior mean deviance, $\overline{D(\Theta)}$, can be estimated by averaging $D(\Theta)$ over all of the steps in the chain, and $D(\tilde{\Theta})$ simply requires an estimator of Θ, such as the posterior mean.

Extension of DIC to missing data models

With data-augmented models, the likelihood function $f(\mathbf{y}|\Theta)$ is often not available in closed form, because it depends on missing data $\mathbf{z}$ as well as the observed data $\mathbf{y}$. In this case, $f(\mathbf{y}|\Theta)$ is called as the *observed* likelihood, while $f(\mathbf{y},\mathbf{z}|\Theta)$ is termed the *complete* likelihood.

The DIC for a missing data model can therefore be re-written in terms of the complete likelihood, as follows:

$$\text{DIC} = -4E_{\Theta}[\log f(\mathbf{y},\mathbf{z}|\Theta)|\mathbf{y},\mathbf{z}] + 2\log f(\mathbf{y},\mathbf{z}|E_{\Theta}[\Theta|\mathbf{y},\mathbf{z}])$$

Since the data $\mathbf{z}$ are, by definition, missing, this quantity can not be computed directly; however, if the distribution of $\mathbf{Z}$ is known or can be approximated (e.g. using a data augmentation MCMC algorithm), it is sufficient to take the expectation of DIC with respect to $\mathbf{Z}$:

$$\text{DIC} = E_{\mathbf{Z}}[\text{DIC}(\mathbf{y},\mathbf{Z})|\mathbf{y}]$$

$$= -4E_{\Theta,\mathbf{Z}}[\log f(\mathbf{y},\mathbf{Z}|\Theta)|\mathbf{y}] + 2E_{\mathbf{Z}}[\log f(\mathbf{y},\mathbf{Z}|E_{\Theta}[\Theta|\mathbf{y},\mathbf{Z}])|\mathbf{y}]$$

The first term in this expression can be estimated using the posterior distributions of Θ and $\mathbf{Z}$ from a data augmentation MCMC algorithm, but the second term requires calculation of the posterior mean $E_{\Theta}[\Theta|\mathbf{y},\mathbf{Z}]$ for each value of $\mathbf{Z}$, which is inconvenient. However, this term can be reformulated to make estimation more straightforward.
Recall that the formula for DIC is as follows:

$$\text{DIC} = 2\overline{D(\Theta)} - D(\tilde{\Theta})$$

And recall that, if we set $h(\mathbf{y}) = 1$, the formula for deviance simplifies to

$$D(\Theta) = -2\log f(\mathbf{y}|\Theta)$$

and therefore the second term in the DIC expression can be rewritten as

$$D(\tilde{\Theta}) = -2\log f(\mathbf{y}|\tilde{\Theta})$$

Expectation-maximization (EM) algorithms suggest a way to approximate the log-likelihood in the context of missing data:

$$\log f(\mathbf{y}|\Theta) = E_{\mathbf{Z}}[\log f(\mathbf{y},\mathbf{Z}|\hat{\Theta}(\mathbf{y}))|\mathbf{y},\hat{\Theta}(\mathbf{y})]$$

where $\hat{\Theta}(\mathbf{y})$ is an estimator of Θ based on the observed data $\mathbf{y}$.

Thus, an alternative way of writing DIC is as follows:

$$\text{DIC} = -4E_{\Theta,\mathbf{Z}}[\log f(\mathbf{y},\mathbf{Z}|\Theta)|\mathbf{y}] + 2E_{\mathbf{Z}}[\log f(\mathbf{y},\mathbf{Z}|\hat{\Theta}(\mathbf{y}))|\mathbf{y},\hat{\Theta}(\mathbf{y})]$$

The second term in this expression can be estimated by running a second MCMC with the parameters Θ fixed at $\hat{\Theta}(\mathbf{y})$ and taking the expectation of the log-likelihood with respect to the posterior distribution of the missing data $\mathbf{Z}$.

Part II: Application

We now show how DIC is calculated for the models of pre-symptomatic transmission described in the Materials & Methods. Recall that the generation interval τ is assumed to follow a gamma distribution with shape parameter α and rate parameter β (incubation-independent model) or β/θ_1 (incubation-dependent model). The incubation periods of the infector and infectee are denoted θ_1 and θ_2, respectively, while δ refers to the serial interval.

We used an MCMC algorithm with data augmentation to estimate the parameters α and β, as well as the missing data $\boldsymbol{\theta_1}$ and $\boldsymbol{\theta_2}$, by fitting to the serial interval data $\boldsymbol{\delta}$. Thus, in the notation of Part 1, the serial interval data $\boldsymbol{\delta}$ are the observed data $\mathbf{y}$, the incubation periods $\boldsymbol{\theta_1}$ and $\boldsymbol{\theta_2}$ comprise the missing data $\mathbf{z}$, and the parameters α and β are the model parameters Θ.

At the end of Part 1, we arrived at the following expression for DIC for a missing data model:

$$\text{DIC} = -4E_{\Theta,\mathbf{Z}}[\log f(\mathbf{y},\mathbf{Z}|\Theta)|\mathbf{y}] + 2E_{\mathbf{Z}}[\log f(\mathbf{y},\mathbf{Z}|\hat{\Theta}(\mathbf{y}))|\mathbf{y},\hat{\Theta}(\mathbf{y})]$$

Rewriting this for our models, we get:

$$\text{DIC} = -4E_{\alpha,\beta,\boldsymbol{\theta_1},\boldsymbol{\theta_2}}[\log f(\boldsymbol{\delta},\boldsymbol{\theta_1},\boldsymbol{\theta_2}|\alpha,\beta)|\boldsymbol{\delta}] + 2E_{\boldsymbol{\theta_1},\boldsymbol{\theta_2}}[\log f(\boldsymbol{\delta},\boldsymbol{\theta_1},\boldsymbol{\theta_2}|\hat{\alpha},\hat{\beta})|\boldsymbol{\delta},\hat{\alpha},\hat{\beta}]$$

From this point on, since our focus is application rather than theory, we drop several redundant conditionals from the equations to improve readability. This simplifies this above to

$$\text{DIC} = -4E_{\alpha,\beta,\boldsymbol{\theta_1},\boldsymbol{\theta_2}}[\log f(\boldsymbol{\delta},\boldsymbol{\theta_1},\boldsymbol{\theta_2}|\alpha,\beta)] + 2E_{\boldsymbol{\theta_1},\boldsymbol{\theta_2}}[\log f(\boldsymbol{\delta},\boldsymbol{\theta_1},\boldsymbol{\theta_2}|\hat{\alpha},\hat{\beta})]$$

The first component of this expression can be rewritten as follows:

$$-4E_{\alpha,\beta,\boldsymbol{\theta_1},\boldsymbol{\theta_2}}[\log f(\boldsymbol{\delta},\boldsymbol{\theta_1},\boldsymbol{\theta_2}|\alpha,\beta)]$$

$$= -\frac{4}{M}\sum_{i=1}^{M}\log f(\boldsymbol{\delta},\boldsymbol{\theta_1}^{(i)},\boldsymbol{\theta_2}^{(i)}|\alpha^{(i)},\beta^{(i)})$$

$$= -\frac{4}{M}\sum_{i=1}^{M}\sum_{k=1}^{N}\log f(\delta_k,\theta_{1(k)}^{(i)},\theta_{2(k)}^{(i)}|\alpha^{(i)},\beta^{(i)})$$

$$= -\frac{4}{M}\sum_{i=1}^{M}\sum_{k=1}^{N}\log f(\delta_k|\theta_{1(k)}^{(i)},\theta_{2(k)}^{(i)},\alpha^{(i)},\beta^{(i)}) + \log f_\theta(\theta_{1(k)}^{(i)}) + \log f_\theta(\theta_{2(k)}^{(i)})$$

$$= -\frac{4}{M}\sum_{i=1}^{M}\sum_{k=1}^{N} \log f_\tau(\delta_k + \theta_{1(k)}^{(i)} - \theta_{2(k)}^{(i)}|\alpha^{(i)}, \beta^{(i)}) + \log f_\theta(\theta_{1(k)}^{(i)}) + \log f_\theta(\theta_{2(k)}^{(i)})$$

where M is the number of iterations in the thinned converged Markov chain, with a superscript $^{(i)}$ denoting the ith iteration; N is the number of infector-infectee pairs represented in the serial interval data set, with a subscript $_{(k)}$ denoting the kth pair; f_τ is the generation interval distribution (with parameters α and β); and f_θ is the prior for the incubation period.

The second component of the expression can similarly be rewritten:

$$2E_{\boldsymbol{\theta}_1,\boldsymbol{\theta}_2}[\log f(\boldsymbol{\delta}, \boldsymbol{\theta}_1, \boldsymbol{\theta}_2|\hat{\alpha}, \hat{\beta})]$$

$$= \frac{2}{M'}\sum_{i=1}^{M'} \log f(\boldsymbol{\delta}, \boldsymbol{\theta}_1{'}^{(i)}, \boldsymbol{\theta}_2{'}^{(i)}|\hat{\alpha}, \hat{\beta})$$

$$= \frac{2}{M'}\sum_{i=1}^{M'}\sum_{k=1}^{N} \log f(\delta_k, \theta_{1(k)}{'}^{(i)}, \theta_{2(k)}{'}^{(i)}|\hat{\alpha}, \hat{\beta})$$

$$= \frac{2}{M'}\sum_{i=1}^{M'}\sum_{k=1}^{N} \log f(\delta_k|\theta_{1(k)}{'}^{(i)}, \theta_{2(k)}{'}^{(i)}, \hat{\alpha}, \hat{\beta}) + \log f_\theta(\theta_{1(k)}{'}^{(i)}) + \log f_\theta(\theta_{2(k)}{'}^{(i)})$$

$$= \frac{2}{M'}\sum_{i=1}^{M'}\sum_{k=1}^{N} \log f_\tau(\delta_k + \theta_{1(k)}{'}^{(i)} - \theta_{2(k)}{'}^{(i)}|\hat{\alpha}, \hat{\beta}) + \log f_\theta(\theta_{1(k)}{'}^{(i)}) + \log f_\theta(\theta_{2(k)}{'}^{(i)})$$

where $\hat{\alpha}$ and $\hat{\beta}$ are the parameter estimates (in this case, posterior means) from the original MCMC, and apostrophes indicate quantities from a secondary MCMC in which α and β are fixed at $\hat{\alpha}$ and $\hat{\beta}$, respectively.

References

1. Lauer SA, Grantz KH, Bi Q, et al. The Incubation Period of Coronavirus Disease 2019 (COVID-19) From Publicly Reported Confirmed Cases: Estimation and Application. Ann Intern Med. 2020;172(9):577-82. doi:10.7326/M20-0504
2. Zhang J, Litvinova M, Wang W, et al. Evolving epidemiology and transmission dynamics of coronavirus disease 2019 outside Hubei province, China: a descriptive and modelling study. Lancet Infect Dis. 2020. doi:10.1016/S1473-3099(20)30230-9
3. Backer JA, Klinkenberg D, Wallinga J. Incubation period of 2019 novel coronavirus (2019-nCoV) infections among travellers from Wuhan, China, 20-28 January 2020. Euro Surveill. 2020;25(5). doi:10.2807/1560-7917.ES.2020.25.5.2000062
